# Perceived Empowering Leadership, Harmonious Passion, and Employee Voice: The Moderating Role of Job Autonomy

**DOI:** 10.3389/fpsyg.2019.01484

**Published:** 2019-07-16

**Authors:** Ang Gao, Jing Jiang

**Affiliations:** ^1^School of Management and Economics, Beijing Institute of Technology, Beijing, China; ^2^School of Tourism Sciences, Beijing International Studies University, Beijing, China

**Keywords:** empowering leadership, voice, harmonious passion, job autonomy, self-determination theory

## Abstract

Based on self-determination theory, we conceptualized the effect of empowering leadership on employee voice *via* harmonious passion. This paper further explored the moderating effect of job characteristics in the voice process and proposed a moderated mediation model. Using a sample of 674 supervisor–subordinate dyads, we found that (1) empowering leadership was positively related to employees’ harmonious passion and voice behavior, (2) harmonious passion played a mediating role in the relationship between empowering leadership and employee voice, and (3) job autonomy strengthened the effect of harmonious passion on employee voice, which, in turn, enhanced the mediated relationship between empowering leadership and employee voice *via* harmonious passion. We discuss the implications of these findings for research and practice.

## Introduction

Employee voice refers to the expression of constructive opinions, concerns, or ideas about work-related issues (Van Dyne et al., 2003). As a source of information, employee voice has been recognized as a valuable input that serves organizational development. However, employees are not always willing to share their thoughts or ideas, especially when doing so goes beyond their duties or probably brings some undesirable outcomes (Detert and Edmondson, 2011).

Given the challenge-oriented and risky nature of voice behavior, the voice process is usually delineated as a rational calculation in which employees weigh instrumentality and potential risk (Morrison, 2014). Recent decades have seen a rapid growth in the number of studies exploring the psychological factors that help employees overcome the fear of voice, such as psychological safety (Liang et al., 2012), voice efficacy (Jiang et al., 2018), and psychological empowerment (Raub and Robert, 2012). Morrison (2014) argued that conscious processing is only part of the voice story. Considering that voice is inherently discretionary, employees need more autonomous motivation and a greater perception of self-interest to speak up for improvement (LePine and Van Dyne, 1998; Morrison and Phelps, 1999; Liu et al., 2010; Detert and Edmondson, 2011; Morrison, 2014). Harmonious passion has been theorized and confirmed as an autonomous motivation (Vallerand et al., 2003; Vallerand and Miquelon, 2007; Liu et al., 2011; Luh and Lu, 2012) by which individuals freely and volitionally view work as important to their identities because of characteristics of the work itself (Ho et al., 2018, p. 114). If employees’ passion for their task is harmonious, they will autonomously promote optimal functioning and protect against poor functioning (Keyes, 2007). Employees will dare to proactively challenge the status quo and make constructive suggestions if their job is enjoyable. In this vein, harmonious passion is an important autonomous motivation to stimulate employees to voice their thoughts that has been ignored in the previous literature.

Although harmonious passion plays a key role in triggering employee voice, we know little about what factors can promote one’s harmonious passion and further drive him or her to speak up. Among various contextual factors, leadership is a critical factor that motivates subordinates to express their opinions. The previous literature presents three major approaches to examining the influence of leaders on employee voice. The first approach focuses on the characteristics of leaders that make employees voice their thoughts, such as leader openness (Detert and Burris, 2007). The second approach emphasizes how the relationships between leaders and subordinates influence employee voice (e.g., Van Dyne et al., 2008). The third approach explores the effect of leader behavior on employee voice, including transformational leadership, ethical leadership, authentic leadership, and abusive supervision (Hsiung, 2012; Farh and Chen, 2014; Chen and Hou, 2016; Duan et al., 2017). Empowering leadership can enhance the motivation and autonomy perception of employees in their work (Seibert et al., 2004, 2011; Zhang and Bartol, 2010), and it appears desirable for empowering leaders to endow their employees with autonomy to challenge the status quo and make constructive changes. Thus, empowering leaders can be treated as contextual autonomy support to trigger employees’ autonomous motivation (such as harmonious passion), which in turn inspires employees to voice their thoughts (Seibert et al., 2004, 2011; Zhang and Bartol, 2010). In addition, a number of studies have shown that empowering leaders may produce important desired outcomes, such as employee creativity (e.g., Zhang and Bartol, 2010), service performance (e.g., Wallace et al., 2011), and turnover behavior (e.g., Chen et al., 2011). Surprisingly, little effort has been made to examine the influence of empowering leadership on employee voice, with the exception of Raub and Robert’s (2012) work in the hospitality industry. The present research proposes that empowering leadership can stimulate employees’ harmonious passion to make them break the silence.

Moreover, we further examine the conditional effect of empowering leadership on employee voice based on self-determination theory. In the workplace, job autonomy is often used to assess situational strength (Fuller et al., 2010; Wang and Cheng, 2010), and it provides employees with freedom from external control in a certain way (Ryan and Deci, 2000). From this perspective, we examine the extent to which “situational forces,” such as job autonomy, might enhance the positive relationship between harmonious passion and voice behavior, which, in turn, will moderate the indirect effect of empowering leadership on voice *via* harmonious passion.

In summary, in this research, we contribute to the literature on empowering leadership and voice in three fundamental ways. First, we theorize the mediating effect of harmonious passion in the relationship between leadership and employee voice. This mediating model captures the continuous process of one’s motivation transformation, and it successfully overcomes the limitation of the dichotomized motivational (intrinsic vs. extrinsic) approach in explaining employee behavior. Second, we adopt self-determination theory to link leadership with employee voice behavior. This research provides an alternative theoretical perspective to understand how empowering leadership influences voice that supplements the study by Raub and Robert (2012). Third, we explore the conditional effect of job autonomy, highlighting its importance in changing the impact of empowering leadership on employee voice behavior. The moderated mediation test reveals a synthesized effect of leader behavior and job characteristics on employee voice.

### Theoretical Background and Hypotheses

As one dominant theory of motivation, self-determination theory postulates two forms of motivation. Intrinsic motivation refers to the state in which one engages in an activity for its own sake of enjoyment, whereas extrinsic motivation refers to the opposite state, in which one engages in an activity for some instrumental reason or goal (Deci and Ryan, 2000; Ryan and Deci, 2000). This dichotomized perspective of motivation has encountered many problems in explaining organizational behavior due to its simplicity (Gagné and Deci, 2005). According to self-determination theory, there are four types of external motivation—external regulated, introjected regulated, identified regulated, and integrated regulated motivation—that vary in their degree of internalization. In other words, the simple dichotomy between extrinsic and intrinsic motivation makes it difficult to reflect the controlled-to-autonomous continuum. As one corollary of self-determination theory, Vallerand et al. (2003) postulated that harmonious passion could reflect the extent to which the activity has been autonomously internalized into one’s identity. Harmonious passion is superior to the dichotomized (intrinsic vs. extrinsic) motivation in connecting contextual autonomy support with proactive employee behaviors (Liu et al., 2011). Thus, we conceptualize harmonious passion as a mechanism of autonomous motivation that mediates the relationship between contextual autonomy support (empowering leadership) and individual discretionary behavior (employee voice).

### Employees’ Harmonious Passion and Voice Behavior

According to how a passionate activity is internalized in one’s core self or identity, two types of passion, harmonious and obsessive, can be distinguished (Vallerand et al., 2003). Harmonious passion results from an autonomous internalization of an activity in a person’s identity (Vallerand et al., 2003, p. 757), which produces a motivational force to willingly engage in the activity. By contrast, obsessive passion results from a controlled internalization of an activity in one’s identity; it originates from interpersonal pressure because certain contingencies are attached to the activity (Vallerand et al., 2003, p. 757). This research mainly focuses on harmonious passion because it is more consistently associated with the discretionary characteristic of voice (Liu et al., 2010, 2011; Ho et al., 2018). Harmonious passion provides a better angle from which to understand the effect of autonomous motivation on employee voice.

Harmonious passion is composed of affective and cognitive elements. The affective component reflects the extent to which individuals love their jobs. The cognitive element refers to the recognition of the importance of their work (Ho et al., 2018). Given the two components of harmonious passion, we conclude that employees’ harmonious passion can promote employee voice behavior. First, harmonious passion indicates that an individual is joyfully engaged in an activity because in his/her heart, he/she loves the activity (Vallerand et al., 2003, 2007; Vallerand and Miquelon, 2007; Ho et al., 2018). If employees have harmonious passion for their work, then they will treat the job like it is their “own.” Driven by inner passion, employees will put great effort into their work and have a strong sense of mission to make constructive suggestions or point out problems to improve their work. Second, harmonious passion stimulates employees to invest more cognitive energy at work (Ho et al., 2011, 2018). Employees with harmonious passion can perceive the significance of their jobs and consider their work important to their identities (Ho et al., 2018). They are willing to share ideas to improve their work to achieve their work goals. Moreover, a positive relationship has been shown between harmonious passion and change-oriented behaviors, such as employee creativity (Liu et al., 2011; Luh and Lu, 2012). It is reasonable to suggest that employees tend to engage in extra role behavior, such as giving advice, when they have harmonious passion for their work.

Hypothesis 1: Employees’ harmonious passion is positively related to voice behavior.

### Perceived Empowering Leadership and Employees’ Harmonious Passion

Perceived empowering leadership is positively related to employees’ harmonious passion for several reasons. Empowering leaders value their employees’ autonomy (Ahearne et al., 2005) and prefer to energize their subordinates by sharing power. With autonomous support, employees feel released from bureaucratic constraints and are usually willing to invest their energy in tasks in which they are truly interested, thereby leading to passion for their job (Vallerand et al., 2003; Liu et al., 2011). The central feature of harmonious passion is one’s identity. Social psychological research shows that the autonomy support that an individual receives from his/her important relationships facilitates a deeper autonomous internalization of activities in his/her identity (Mageau et al., 2009; Liu et al., 2011). Empowering leaders endow their employees with autonomy, which, to some extent, accelerates the internalization of tasks into employees’ identity and promotes their harmonious passion. Moreover, empowering leaders highlight the significance of the work (Zhang and Bartol, 2010; Seibert et al., 2011). When employees perceive that they are pursuing meaningful, shared objectives through clear processes that have been outlined by the leaders, they are more likely to develop harmonious passion for their work (Vallerand et al., 2003). Based on the above analysis, we propose the following:

Hypothesis 2: Perceived empowering leadership is positively related to employees’ harmonious passion.

### Perceived Empowering Leadership and Employee Voice

Empowering leadership emphasizes power-sharing behaviors oriented toward enhancing the autonomous motivation of subordinates, and it involves enhancing work meaningfulness, promoting participation, expressing confidence, and providing autonomy (Conger and Kanungo, 1988; Ahearne et al., 2005; Zhang and Bartol, 2010). According to self-determination theory, empowering leaders can be treated as contextual support that triggers employees’ autonomous motivation, which, in turn, will inspire employees to voice their thoughts (Seibert et al., 2004, 2011; Zhang and Bartol, 2010). Self-determination theory posits that people continually seek to satisfy basic psychological needs—such as the need for autonomy—to experience ongoing personal growth and well-being (Deci and Ryan, 2000; Ryan and Deci, 2000). The need for autonomy refers to people’s need to believe that they choose their own actions, such as initiating, regulating, and maintaining their own behavior (Deci and Ryan, 2000; Ryan and Deci, 2000), and people experience a personal sense of freedom when this need is met (Deci and Ryan, 2000; Ryan and Deci, 2000). Based on self-determination theory, when people experience a sense of choice and volition, they are likely to challenge the status quo through voice behavior (Deci and Ryan, 2000).

There is a solid theoretical rationale for the contention that perceived empowering leadership is positively associated with employee voice behavior. For instance, empowering leaders convey the value and importance of work to employees, which helps their subordinates experience high potency in performing their tasks and thus devise more constructive suggestions (Zhang and Bartol, 2010; Raub and Robert, 2012). Similarly, empowering leaders encourage the sharing of ideas and opinions on collective decision making. Employees are allowed more leeway in communicating and challenging the status quo (Raub and Robert, 2010, 2012; Zhang and Bartol, 2010; Hon and Chan, 2012), leading to more voice behavior. Moreover, empowering leaders express confidence in their employees’ performance and give fair consideration to the ideas presented, which helps promote a strong sense of competence among work members. Employees are more likely to focus on tasks rather than worry and be diffident; thus, they become willing to take risks to express their ideas (Raub and Robert, 2012). In addition, empowering leaders provide autonomy, which allows people to engage in their work. When they have problems or concerns in the work setting, they will be willing to give voice to their thoughts to carry out adjustments rather than passively keep silent. The previous literature also shows that empowering leadership can predict employee voice and challenge behaviors (Raub and Robert, 2010, 2012; Zhang and Bartol, 2010; Hon and Chan, 2012). According to the foregoing analysis, this study proposes the following:

Hypothesis 3: Perceived empowering leadership is positively related to employee voice.

### The Mediating Role of Harmonious Passion Between Empowering Leadership and Voice

In line with the above analysis, we argue that perceived empowering leadership exerts a positive effect on employees’ harmonious passion and further influences employee voice behavior. Drawing on self-determination theory, empowering leadership can be treated as some contextual support that endows employees with the leeway to engage in their favorite work (Zhang and Bartol, 2010; Seibert et al., 2011). Subordinates are trusted and respected and have enough authority to handle their own affairs (Zhang and Bartol, 2010). Leaders’ empowering behaviors not only enhance employees’ liking for their work but also promote their awareness of the meaning of their jobs (Ho et al., 2011, 2018). Accordingly, employees’ harmonious passion (autonomous motivation) will be triggered. Passionate employees internalize their work as part of their identity, and they tend to perform risky behaviors such as voicing their thoughts to improve their work (Ho et al., 2011, 2018). Although no empirical research has tested harmonious passion as a mediator of the relationship between empowering leadership and employee voice behavior, previous studies have demonstrated that harmonious passion plays a mediating role between contextual support for autonomy and the job creativity of team members (Liu et al., 2011). Thus, we integrate the previous theoretical arguments as a test of the overall hypothetical model of the current study and hypothesize the following:

Hypothesis 4: Harmonious passion mediates the relationship between empowering leadership and employee voice behavior.

### The Moderating Role of Job Autonomy

Job autonomy refers to the degree to which the job provides employees with substantial freedom, independence, and discretion in scheduling their work and in determining the procedures to be used in carrying it out (Hackman and Oldham, 1975, p. 165). Job autonomy sets people free from external constraints and regulations (Deci et al., 1989; Spreitzer, 1995). In other words, motivated employees can undertake their own planning in such conditions. Therefore, we further theorize that job autonomy can strengthen the relationship between employees’ harmonious passion and voice behavior, which, in turn, will enhance the indirect effect of empowering leadership on employee voice *via* harmonious passion.

Employees with high job autonomy are liberated from bureaucracy (Wang and Cheng, 2010; Dhar, 2016). They usually have the right to make their own decisions in their daily work. Consequently, passionate employees with high job autonomy do not have to deal with various work restrictions. They have more leeway to express their ideas to improve their work. In addition, an increase in job autonomy allows employees to engage in their “own” enjoyable work (Dhar, 2016). If passionate employees have freedom in a job that they love, they will invest great effort in identifying problems and generating ideas for work improvement. Moreover, employees with high job autonomy will be more likely to engage in creative processes such as optimizing work flow or improving work efficiency (Oldham and Cummings, 1996; Tierney and Farmer, 2002; Wang and Cheng, 2010). Under this condition, employees who are full of harmonious passion for their work will tend to put forward advice for better performance. Contrarily, a job with low autonomy does not encourage employees to perform risky behaviors (Wang and Cheng, 2010), and the effect of employees’ harmonious passion on voice behavior will be weakened due to uncontrollable external factors such as bureaucratic constraints.

In situations where employees have a high degree of job autonomy, empowering leaders endow their employees with a large amount of leeway to pursue their enjoyable work, which, to a large extent, meets subordinates’ inner needs (Liu et al., 2011). Employees with strong harmonious passion will try their best to make the job perfect, even challenging the status quo to offer constructive ideas. By contrast, low job autonomy limits the freedom of employees and constrains employees’ power in decision making (Hackman and Oldham, 1975; Man and Lam, 2003). What empowering leaders advocate is inconsistent with the characteristics of low-autonomy work (Lee et al., 2018). Thus, when job autonomy is low, empowering leaders are likely to play little or no role in stimulating their employees’ voice *via* harmonious passion. Therefore, we propose the following:

Hypothesis 5a: Job autonomy moderates the relationship between employees’ harmonious passion and voice behavior, such that the relationship is stronger when job autonomy is higher rather than lower.

Hypothesis 5b: Job autonomy moderates the indirect relationship between empowering leadership and employee voice behavior via harmonious passion, such that the relationship is stronger when job autonomy is higher rather than lower.

## Materials and Methods

### Sample and Procedure

We conducted a survey in a large energy group in mainland China. Before administering our survey, we explained the purpose of this study. Employees were told that participation is voluntary, and we cordially invited them to participate in our study. We also ensured confidentiality by indicating that no one from the company would see anyone’s individual responses and that all data would be used only for purposes of academic research. This study was carried out in accordance with the recommendations of the ethics committee of Beijing International Studies University. All participants gave written informed consent in accordance with the Declaration of Helsinki. The protocol was approved by the ethics committee of Beijing International Studies University.

We distributed the questionnaire to 840 supervisor–subordinate dyads, and the final sample consisted of 674 supervisor–subordinate dyads, yielding a valid response rate of 80.24%. Across the subordinate sample, employees with a bachelor’s degree or higher accounted for 47.6%, and the average age of employees was 30.41 (*SD* = 7.23; range = 18–60).

### Measures

All measures used in the current research are Chinese versions validated in studies previously conducted in a Chinese context. The subordinates completed the measures for empowering leadership, harmonious passion, and job autonomy; employee voice was rated by their supervisors. All measures used a five-point response format ranging from “strongly disagree” (1) to “strongly agree” (5).

#### Empowering Leadership

Empowering leadership was measured by the 12-item scale developed by Ahearne et al. (2005), which is validated and the most frequently used in empowering leadership research (Lee et al., 2018). Sample items include the following: “my manager helps me understand how my objectives and goals relate to those of the company” and “my manager helps me understand the importance of my work to the overall effectiveness of the company.” Cronbach’s alpha for this measure is 0.89.

#### Harmonious Passion

Harmonious passion was measured by the seven-item scale developed by Liu et al. (2011). Sample items include the following: “the new things that I discover with my job allow me to appreciate it even more” and “my job is in harmony with the other activities in my life.” Cronbach’s alpha for this measure is 0.77. To capture a holistic view of employee passion, we also measured obsessive passion (Liu et al., 2011). Cronbach’s alpha for this measure is 0.72.

#### Job Autonomy

We used the three-item job autonomy scale developed by Hackman and Oldham (1975). The following is a sample item included in the scale: “I have considerable opportunity for independence and freedom in how I do my job.” Cronbach’s alpha for this measure is 0.77.

#### Employee Voice

To measure employee voice, we used the six-item voice scale developed by LePine and Van Dyne (1998). The following is a sample item included in the scale: “this employee speaks up with ideas for new projects or changes in procedures.” Cronbach’s alpha for this measure is 0.92.

#### Control Variables

##### Controls

To demonstrate the predictive validity of empowering leadership for employee voice, in this study, we controlled for supervisors’ transformational leadership. In addition, we controlled for the demographic variables of the leaders and the employees, including leader gender, leader age, subordinate gender, subordinate age, and subordinate education. We also controlled for obsessive passion and psychological safety (Liang et al., 2012) while examining the mediating and moderated mediating effects.

## Results

Table 1 presents the means, standard deviations, and bivariate correlations for all variables. As expected, empowering leadership was positively correlated with employees’ harmonious passion (*r* = 0.38, *p* < 0.01) and employee voice (*r* = 0.16, *p* < 0.01). Before testing our hypotheses, we conducted a series of confirmatory factor analyses (CFAs) to examine the distinctiveness of our study variables. The results of CFA, conducted using LISREL 8.51, show that the six-factor model (i.e., empowering leadership, harmonious passion, obsessive passion, psychological safety, job autonomy, and employee voice) fit better [χ^2^ = 2185.84, df = 650, *p* < 0.01; root mean square error of approximation (RMSEA) = 0.06, comparative fit index (CFI) = 0.87, root mean square residual (RMR) = 0.04] than the one-factor model (χ^2^ = 10,166.98, df = 665, *p* < 0.01; RMSEA = 0.15, CFI = 0.41, RMR = 0.11) or any other five-factor models (chi-square changes ranged from 515.19 to 4069.47, Δdf = 5, *p* < 0.01).

**TABLE 1:** Descriptive statistics, correlations, and reliabilities.

	**Mean**	***SD***	**1**	**2**	**3**	**4**	**5**	**6**	**7**	**8**	**9**	**10**	**11**
(1) Subordinate gender	–	–											
(2) Subordinate age	30.40	7.22	–0.01										
(3) Subordinate education	–	–	–0.08	–0.02^∗∗^									
(4) Leader gender	0.22	0.41	0.27^∗∗^	0.05	0.02								
(5) Leader age	33.70	7.69	0.01	0.54^∗∗^	0.00	0.15^∗∗^							
(6) Empowering leadership	3.86	0.56	–0.03	0.07	0.14^∗∗^	0.07	0.09^*^	(0.89)					
(7) Harmonious passion	4.06	0.51	–0.05	–0.07	0.17^∗∗^	0.13^∗∗^	–0.07	0.38^∗∗^	(0.77)				
(8) Obsessive passion	2.51	0.60	–0.06	0.12^∗∗^	–0.15^∗∗^	–0.07	0.09^*^	–0.12^∗∗^	–0.10^∗∗^	(0.72)			
(9) Psychological safety	4.26	0.54	–0.06	–0.01	0.15^∗∗^	0.02	–0.00	0.39^∗∗^	0.42^∗∗^	–0.19^∗∗^	(0.84)		
(10) Job autonomy	2.82	0.81	–0.18^∗∗^	0.05	0.23^∗∗^	–0.02	0.05	0.35^∗∗^	0.29^∗∗^	–0.00	0.24^∗∗^	(0.77)	
(11) Employee voice	3.66	0.86	0.03	0.14^∗∗^	0.02	0.13^∗∗^	0.12^∗∗^	0.16^∗∗^	0.15^∗∗^	–0.03	0.09^*^	0.11^∗∗^	(0.92)

*N* = 514–674. Cronbach’s alpha is in parentheses on the diagonal. ^*^p < 0.05, ^∗∗^*p* < 0.01 (two-tailed).

Table 2 summarizes the regression results for testing Hypothesis 1, which predicted that employees’ harmonious passion is positively related to employee voice, and Hypotheses 2 and 3, which predicted that perceived empowering leadership is positively related to employee’s harmonious passion and voice. After controlling for obsessive passion, psychological safety, and demographic variables, we found a significant effect of harmonious passion on employee voice (β = 0.16, *p* < 0.01). In addition, we found that perceived empowering leadership had significant effects on employee’s harmonious passion (β = 0.32, *p* < 0.01) and voice (β = 0.20, *p* < 0.01) after controlling for supervisors’ transformational leadership and the demographic variables. Thus, Hypotheses 1–3 were all supported.

**TABLE 2:** Regression results for Hypotheses 1–6.

**Models**	**Variables**	***B***	***SE***	***t***	***R*^2^**
Model 1. Voice regressed on harmonious passion	Harmonious passion	0.16	0.05	3.20^∗∗^	0.08^∗∗^
Model 2. Harmonious passion regressed on empowering leadership	Empowering leadership	0.32	0.07	4.76^∗∗^	0.21^∗∗^
Model 3. Voice regressed on empowering leadership	Empowering leadership	0.20	0.08	2.72^∗∗^	0.07^∗∗^
Model 4. Voice regressed on empowering leadership and harmonious passion	Empowering leadership	0.14	0.08	1.79	
	Harmonious passion	0.15	0.05	2.75	0.10^∗∗^
Model 5. Voice regressed on harmonious passion, job autonomy, and the interaction term	Harmonious passion	0.12	0.05	2.61^∗∗^	0.11^∗∗^
	^*^job autonomy				
Model 6. Voice regressed on empowering leadership, harmonious passion, job autonomy, and the interaction term	Empowering leadership	0.12	0.08	1.45	
	Harmonious passion	0.14	0.06	2.60^∗∗^	0.12^∗∗^
	Job autonomy	0.05	0.05	0.88	
	Harmonious passion	0.11	0.05	2.35	
	^*^job autonomy				

	**Bootstrap results for indirect effect of empowering leadership on employee voice *via* harmonious passion**				
	
	***ab***	***SE***	**Boot 95% CI**		
	
Empowering leadership	0.04^*^	0.02	[0.01,0.09]		

*N* = 442–476. Standardized regression coefficients are reported. In Model 1 and Model 5, we controlled obsessive passion, psychology safety, and demographic variables including leader age, leader gender, subordinate age, subordinate gender, and subordinate education. In Model 2 and Model 3, we controlled transformational leadership and all demographic variables. In Model 4 and Model 6, we controlled transformational leadership, obsessive passion, psychology safety, and all demographic variables.^*^p < 0.05, ^∗∗^*p* < 0.01 (two-tailed).

Hypothesis 4 predicted that harmonious passion would play a mediating role between perceived empowering leadership and employee voice. To test this indirect effect, we used a bootstrapping approach with the aid of an SPSS macro provided by Preacher and Hayes (2008). As expected, we found that the indirect effect of perceived empowering leadership on employee voice through harmonious passion was.04 (*SE* = 0.02, 95% CI [0.01,0.09]), which supported Hypothesis 4.

**FIGURE 1:**
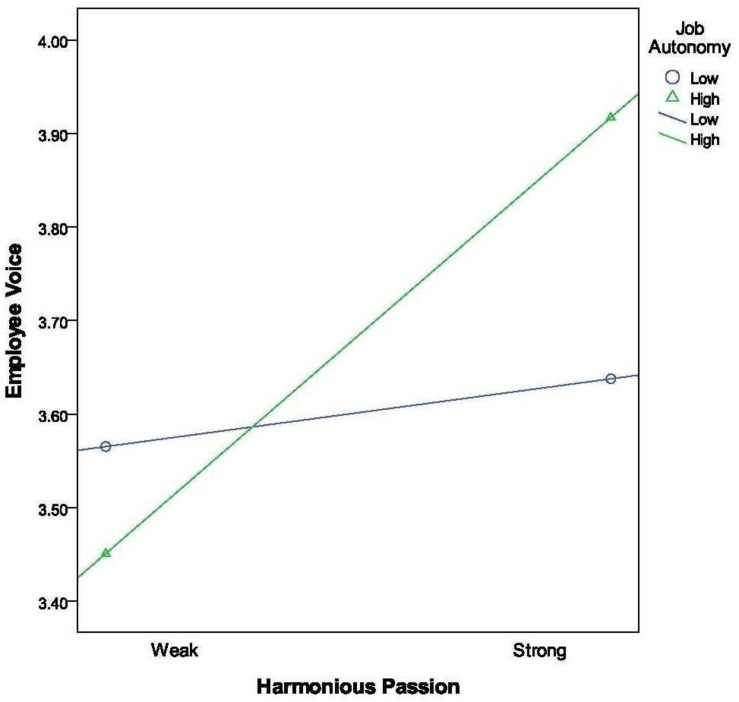
Moderating effect of job autonomy on harmonious passion – employee voice relationship.

Hypothesis 5a focused on the moderating effect of the contextual factor, such that the relationship between harmonious passion and employee voice would be stronger when job autonomy was higher rather than lower. As shown in Table 2, we found that the harmonious passion × job autonomy interaction term is significant (β = 0.12, *p* < 0.01). Following the procedures of Aiken and West (1991), we took the cutoff values of one standard deviation above and below the mean for the relevant variables to obtain four separate plotting points. Figure 1 indicates that job autonomy did indeed strengthen the relationship between harmonious passion and employee voice. Thus, Hypothesis 5 was supported.

Hypothesis 5b predicted that job autonomy would moderate the mediation effect of harmonious passion between perceived empowering leadership and employee voice, such that the mediating effect would be stronger when job autonomy was higher rather than lower. We used the bootstrapping approach provided by the SPSS macro to test the moderated mediating effect. As expected, we found a significant indirect effect of perceived empowering leadership on employee voice (indirect effect: 0.07, *SE* = 0.03, 95% CI [0.03,0.14]) when job autonomy was high and a non-significant indirect effect (indirect effect: 0.01, *SE* = 0.02, 95% CI [−0.03,0.05]) when job autonomy was low. Thus, Hypothesis 5b was supported.

## Discussion

Overall, we found that employees’ harmonious passion not only was related to employee voice but also mediated the relationship between empowering leadership and employee voice behavior. Moreover, job autonomy strengthened the effect of employees’ harmonious passion on employee voice, which, in turn, enhanced the mediated relationship between empowering leadership and employee voice behavior *via* employees’ harmonious passion. Our findings contribute to the literature and management practice.

### Theoretical Implications

First, we explicitly proposed and empirically tested a self-determination theoretical framework to explain the connection of contextual autonomy support with employee voice. The voice process of why employees engage or not has drawn a great deal of attention in the last decade, and more studies have focused on the decision calculus instead of the underlying motivation (Morrison, 2014). More specifically, there is evidence that before performing voice behavior, employees usually become used to calculating the instrumentality and potential risk of doing so (Morrison, 2014). It is necessary to explore the motivational mechanisms of employee voice. We theorized that harmonious passion is a parallel mediator explaining why empowering leadership can enable employee voice. To the best of our knowledge, this research is perhaps the first empirical study to explore harmonious passion as a mediator connecting leadership and employee voice. We also tested the mediating effect of harmonious passion after controlling for employees’ psychological safety. Our findings enrich the voice behavior literature.

Our second contribution is that we have built and tested a conceptual model that uniquely integrates empowering leadership with employee voice based on self-determination theory. Few studies have focused on the influence of empowering leadership on employee voice, except for Raub and Robert’s (2012) work. Our findings make some additional contributions to the literature. On the one hand, we further clarified the underlying theoretical mechanism by pointing out the mediating role of autonomous motivation. On the other hand, we controlled for the effect of transformational leadership and identified the extra effect of empowering leadership on employee voice. Our research extends the findings of Raub and Robert (2012), who highlight the potential value of empowering leadership in stimulating employee voice.

Third, we find that job autonomy plays an important role in how harmonious passion leads to employee voice, which, in turn, influences the indirect effect of empowering leadership on voice *via* harmonious passion. Our research reveals that under conditions of high job autonomy, employees with harmonious passion have more room for work improvement and feel a greater sense of responsibility to express constructive ideas for their work, and vice versa. Furthermore, the characteristic of job autonomy is consistent with empowering leadership’s emphasis on individual self-determination. As highlighted in the preceding section, empowering leaders can trigger employees’ harmonious passion for their work. Additionally, a highly autonomous job design leaves more room for these passionate employees, which further enables and inspires them to voice their concerns. In contrast, under conditions of low job autonomy, the role of empowering leaders in driving employees’ harmonious passion is limited by rule restrictions (Kim et al., 2009), which further weakens the indirect effect of employees’ proactive behaviors. These findings also contribute to the previous literature on job characteristics and voice.

### Managerial Implications

Our theoretical model also has practical implications. First, managers should make the effort to adjust their human resources management practices to stimulate their employees’ work passion. This study introduces the concept of harmonious passion to managers to provide them with an alternative perspective from which to understand employee motivation. We suggest that managers should help their employees become involved and internalize their work in their identities. In regard to autonomous motivation, managers should pay more attention to enhancing employees’ harmonious passion instead of differentiating intrinsic motivation from extrinsic motivation. According to our findings, passionate employees can benefit their organizations through their voice behavior.

Second, managers can empower their employees and encourage them to take the initiative rather than spend their time assigning specific tasks and monitoring their employees’ proactive activities. As shown in our study, empowering leaders can nurture their employees’ harmonious passion, which, in turn, will enhance employee behaviors of giving more constructive suggestions, even after eliminating the interference of supervisors’ transformational behaviors. Leaders’ empowering behaviors appear to be a promising approach for organizations to gain more advice from employees. Thus, organizations can benefit from providing empowering leadership training to managers.

Third, the job design should be an important part of the empowerment strategy. We assume that if a passionate individual can plan his/her own work, then he/she can better understand the job and have more ideas for work improvement. At the same time, he/she may be more willing to give voice to his/her thoughts because of his/her sense of ownership. In contrast, excessive job restrictions may strangle the positive effect of employees’ harmonious passion on voice by depriving employees of initiative. Managers should provide more work autonomy because doing so may enable passionate employees to perform more proactive behaviors.

### Limitations

Our research also has limitations that reveal some valuable directions for future research. First, when examining the mediating effect of harmonious passion, we controlled for obsessive passion and psychological safety but not psychological empowerment. Raub and Robert (2012) found that psychological empowerment could mediate the relationship between empowering leadership and employee voice. We thought that such empowerment could make employees identify with their job and enjoy their work. In other words, psychological empowerment might stimulate employee voice behavior by enhancing employees’ harmonious passion. Although we controlled for the interference of psychological safety, this study did not test the consequential mediating effect. We suggest that future studies should consider psychological empowerment into account.

Second, we investigated the effect of empowering leadership on employee voice behavior at the dyadic level instead of the team level. To avoid common method bias, we invited supervisors to rate employee voice. There might be shared variance among some of the dyads because the employees were nested within their teams. Fortunately, no statistical evidence showed any effect of leadership on employee voice at the group/cross level. However, our sampling strategy might still limit the validity of the research. What’s more, some studies have managed to operationalize empowering leadership at a higher level (e.g., Srivastava et al., 2006; Chen et al., 2007). If future studies can take a team or multilevel perspective, doing so might help to gain a comprehensive understanding of the influence of empowering leadership on employee voice.

The third limitation of this study is that we collected data from a single organization in China, which limits the generalizability of our results. Although the single data source helped in controlling for organization-level confounding variables, future studies should conduct surveys in various companies to increase the external validity of the results. Moreover, previous research has found that culture might moderate the effectiveness of empowering leadership (Cheong et al., 2019). Future studies might take cultural context into consideration while exploring the effect of empowering leadership on employee voice. In addition, this study adopts a cross-sectional design. Future studies may employ longitudinal designs to substantiate the causality of the relationships found.

## Conclusion

This study theorizes the effect of contextual autonomy support (empowering leadership) on individual discretionary behavior (employee voice) *via* autonomous motivation (harmonious passion) and probes the moderating effect of job characteristics (job autonomy). The results show that employees’ harmonious passion mediates the relationship between empowering leadership and employee voice behavior. Employees’ job autonomy strengthens the relationship between their harmonious passion and voice, which, in turn, enhances the indirect effect of empowering leadership on employee voice *via* employees’ harmonious passion.

## Data Availability

All datasets generated for this study are included in the manuscript and/or the supplementary files.

## Author Contributions

AG and JJ together developed the research proposal. JJ contacted the company and supervised the data collection. AG completed the statistical analysis. AG and JJ together wrote the manuscript.

## Conflict of Interest Statement

The authors declare that the research was conducted in the absence of any commercial or financial relationships that could be construed as a potential conflict of interest.
